# Third-Order Nonlinear Optical Properties of Aqueous Silver Sulfide Quantum Dots

**DOI:** 10.1021/acs.jpclett.3c02820

**Published:** 2023-12-06

**Authors:** Marta Gordel-Wójcik, Magdalena Malik, Agnieszka Siomra, Marek Samoć, Marcin Nyk

**Affiliations:** † Faculty of Chemistry, University of Wrocław, 14.p F. Joliot-Curie Street, 50-383 Wrocław, Poland; ‡ Faculty of Chemistry, Wrocław University of Science and Technology, Wyb. Wyspiańskiego 27, 50-370 Wrocław, Poland; § Institute of Advanced Materials, Faculty of Chemistry,Wrocław University of Science and Technology, Wyb. Wyspiańskiego 27, PL-50370 Wrocław, Poland

## Abstract

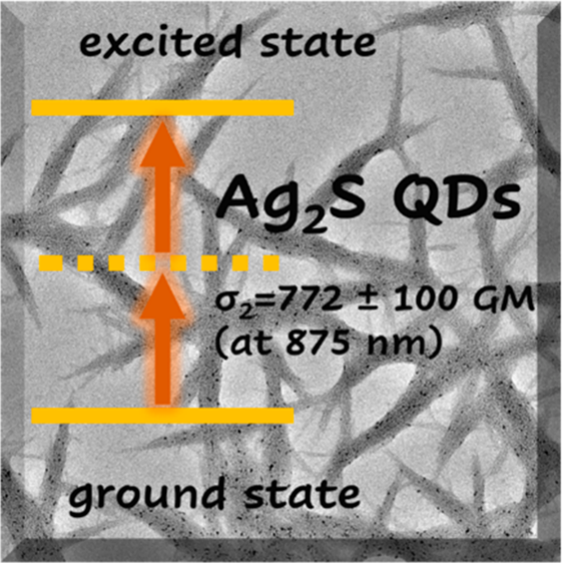

Wide spectral wavelength range (500–1600 nm) measurements of nonlinear optical properties of silver sulfide (Ag_2_S, with 2- or 3-mercaptopropionic acid, 2 or 3MPA ligands) quantum dots (QDs) in aqueous colloidal solutions were performed using the Z-scan technique with tunable ∼55 fs laser pulses at 1 kHz. We have identified regions of the occurrence of various NLO effects including two-photon absorption, nonlinear refraction, as well as saturation of one-photon absorption. At the same time, we evaluated the relationship between the properties of the QDs and the variation of the material that covers their surface. The peak two-photon absorption cross section (σ_2_) values were determined to be 632 ± 271 GM (at 850 nm) for Ag_2_S-2MPA QDs and 772 ± 100 GM (at 875 nm) for Ag_2_S-3MPA QDs. The physicochemical factors influencing the three-dimensional self-organization of Ag_2_S QDs in water as well as their impact on spectroscopic properties were also investigated.

Near-infrared emitting quantum dots (QDs) are required for various nanophotonics and biophotonics applications; however, their most frequently investigated compositions include toxic metals like cadmium, mercury, and lead, which has restricted their applicability. An interesting alternative is provided by QDs of silver compounds, enabling the creation of less hazardous materials. Ag_2_S QDs have attracted significant attention and have been explored in various research endeavors.^1−4^ A recent investigation substantiated the theranostic potential of these compounds through experimentation on mice.^4^ Additionally, it has been verified that only a small amount of silver ions persists after the administration of QDs within the body, a result attributed to the exceedingly low solubility product constant of Ag_2_S QDs (*k*_SO_ = 6.3 × 10^–50^).^4^ Consequently, colloidal silver sulfide QDs hold the potential for being employed in diagnostics and therapeutics with the desired minimization of the risk of metal poisoning.

A specific feature, which is often sought for various nanomaterials, is efficient two-photon absorption (2PA) within the first “optical transmission window” of biological tissues, specifically in the near-infrared (NIR) range (750–1000 nm). The applications of 2PA in this domain are manifold, particularly within optics, imaging, and photomedicine. In the realm of two-photon microscopy, the use of NIR light facilitates deeper tissue penetration due to reduced scattering and absorption.^5^ NIR light with 2PA capabilities can activate photosensitive agents in deeper tissues with high spatial precision, enhancing the efficacy of photodynamic therapy while minimizing harm to adjacent healthy tissues.^6^ QDs with robust 2PA within the NIR spectrum can serve as contrast agents in biological imaging, permitting real-time tracking and study of cellular and molecular processes with enhanced sensitivity and resolution.^7^ Furthermore, the potential of 2PA extends to data storage applications. Materials possessing 2PA characteristics enable nanoscale-level writing, reading, and erasure of data, paving the way for high-density and high-speed data storage devices.^8^

Other nonlinear optical absorption features that a nanomaterial may possess are one-photon absorption saturation and reverse saturation (often the same material may be a two-photon absorber in a certain wavelength range and a saturable absorber or reverse saturable absorber in other ranges; e.g., see ref (9)) that can prove particularly valuable in specific applications relevant to laser systems, such as passive mode-locking and power limiting. Precise knowledge of the nonlinear absorption parameters such as saturation intensity and its dynamics enables the optimization of laser performance and serves to avert harm to vulnerable optical elements.^10^ For instance, what has been notably well elucidated and researched are the novel 2D MXene materials with distinctive optical properties that enable hybrid mode-locking technology and harmonic pulse generation.^11−14^

Our main focus is to present a comprehensive quantitative analysis of the nonlinear optical properties of Ag_2_S QDs colloidal solution across a broad range of wavelengths, with the results being given as the spectra of σ_2_, the one-photon saturable absorption, and the real (refractive) component of the complex hyperpolarizability vs the wavelength. Besides, we examine and clarify the differences in the optical characteristics of Ag_2_S QDs synthesized using 2MPA and 3MPA acids under various conditions. Our investigation delves into the parameters that influence the three-dimensional self-assembly of nanoparticles, while simultaneously striving to elucidate the factors governing this process. We note that self-organization, i.e., the natural arrangement of molecules or nanoparticles into structured patterns without requiring external intervention, can have much importance in the context of 2PA through impacting various aspects of the material’s behavior, such as enhanced absorption,^15,16^ band structure modifications,^17^ or exciton delocalization.^18^

## XRD Patterns

Ag_2_S quantum dots (QDs) coated with 2MPA or 3MPA were synthesized in an aqueous medium. In the initial stage, a mixture of silver salt and either 2MPA or 3MPA was prepared, followed by the introduction of sulfide. Depending on the synthesis conditions, the solution exhibited hues ranging from pale yellow to brown. The stability of the colloidal nanoparticle solutions endured for several months. The crystalline structure of the desiccated QD solutions was examined via powder X-ray diffraction (XRD). Before measurement, the sample underwent a 1 h drying process in nitrogen atmosphere at 180 °C. These conditions led to the decomposition of surface substances (2MPA, 3MPA). The discernible peaks in the corresponding diffraction pattern (Figure S1) can be attributed to the monoclinic α-Ag_2_S XRD pattern (indicated by the red lines in Figure S1, which matches 5JCPDS no. 14-0072).

## Optical Characterization

Subsequently, we examined the optical properties of six distinct colloidal solution samples containing Ag_2_S QDs. The detailed synthesis parameters are provided in Table 1; the absorption spectra are shown in Figure S2; and the corresponding TEM images are depicted in Figures S3, S4, S5, and S6. The shapes of the curves are similar for QDs prepared with either 2MPA or 3MPA acids; they are mostly featureless, an exception being that for the samples synthesized with a Ag-to-S ratio of 6 an absorption band is observed in the near-infrared range, with a maximum located around 850 nm. In syntheses conducted at a temperature of 90 °C, a correlation is evident among the “twin” QDs—those synthesized with identical proportions of reactants and parameters such as pH and temperature, but distinguished by the coating of 2MPA/3MPA. During the initial synthesis stage using equivalent amounts of acids, the pH of the solution with 2MPA was lower by one unit compared to that with 3MPA. This difference in pH had an impact on the size of the resulting QDs, with QDs incorporating 3MPA exhibiting a larger size by approximately 0.3 nm than those coated with 2MPA (see Table 1). An intriguing property of self-organization is demonstrated by QDs synthesized using 3MPA when the Ag:S ratio is 6:1. This phenomenon is evident in TEM images (Figures S5 and S6d,e) and in dynamic light scattering (DLS) measurements (Figure S7), where QDs are observed to aggregate into distinct clusters. However, this self-organization property is not observed in the cases involving the protective coating of 2MPA, or in the case of 3MPA with the Ag:S ratio of 4:1. The probable explanation for these distinct structures lies in the variation of the materials that compose the protective shell of the QDs, as is also evident for the sizes. Yet, this is not the only contributing factor, as self-organization would also occur for Ag:S = 4:1. A crucial role in this process is likely played by an excess of Ag^+^ ions and a pH value exceeding 8. This significance stems from the fact that in systems where self-organization occurs, the pH fluctuates within the range of 7.55–7.65, while in the remaining cases, it assumes values ranging from 8.45 to 8.90. Therefore, this interesting property merits further attention, as, regrettably, no research has been reported in the literature that addresses this particular topic. Hence, to provide more detail, we also involved FT-IR spectroscopy.

**Table 1:** Information about the Synthesis Conditions of Ag_2_S QDs^a^

Sample name	Ag:S	Acid	Temp. (°C)	Reaction time (h)	Size (nm)	Zeta (mV) after the synthesis	Zeta (mV) after 4 weeks	pH after synthesis
**1**	4	2MPA	90	3	2.76 ± 0.81	–11.04	–33.56	8.90
**2**	4	3MPA	90	3	3.03 ± 0.80	–23.90	–32.70	8.80
**3**	6	2MPA	30	1	2.24 ± 0.59	–40.17	–37.69	8.45
**4**	6	3MPA	30	1	2.66 ± 0.60	–38.21	–45.70	7.65
**5**	6	2MPA	90	3	2.58 ± 0.79	–21.56	–37.51	8.50
**6**	6	3MPA	90	3	2.88 ± 0.72	–21.75	–55.47	7.55

a Including variable parameters such as the Ag-to-S ratio, type of acid used, reaction temperature, reaction time, sizes, measured zeta potential of the solution on the day of synthesis and after 4 weeks, as well as the pH of the solution after synthesis and centrifugation.

**Figure 1:**
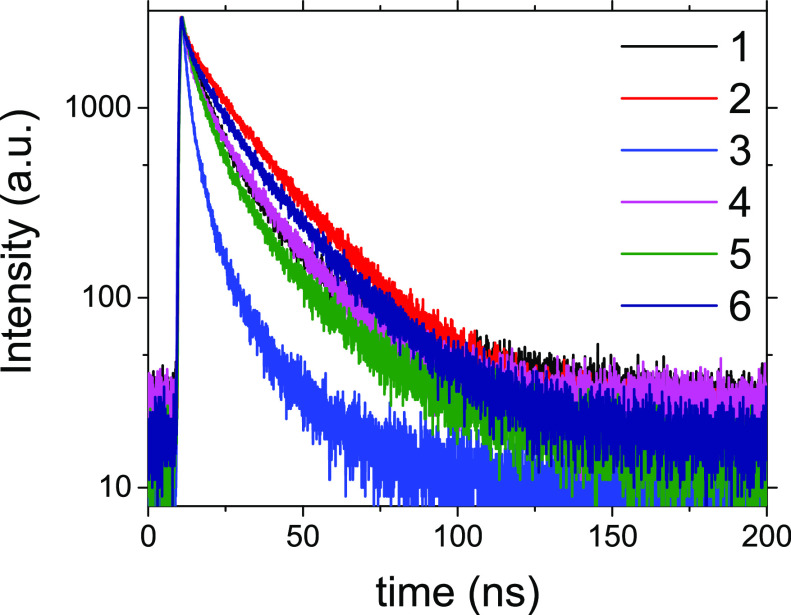
Fluorescence decays of **1**–**6** colloidal solutions of Ag_2_S QDs.

**Figure 2:**
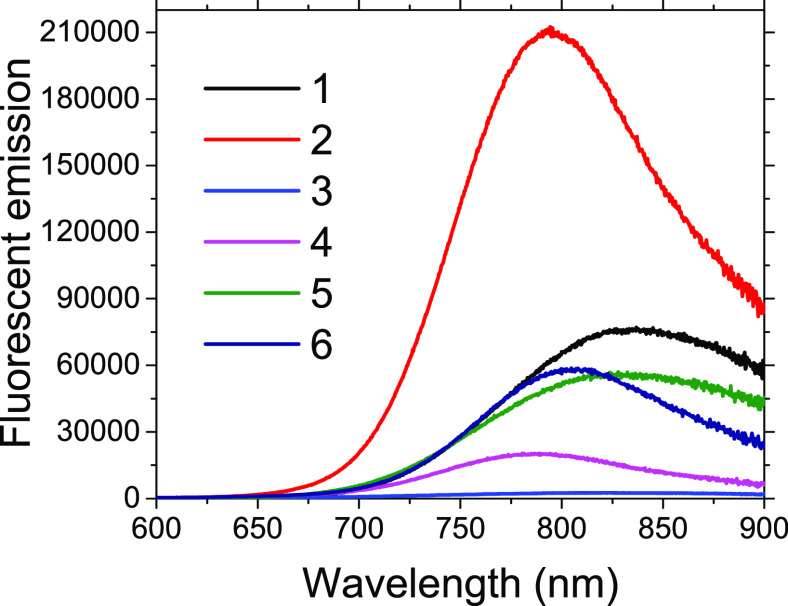
Fluorescence emission spectra of the samples measured under excitation with a xenon lamp at 450 nm. In each of the investigated “twin” syntheses, samples prepared with 3MPA (2, 4, 6) display elevated fluorescence emission levels and narrower emission bands. Conversely, samples synthesized using 2MPA (1, 3, 5) exhibit emissions shifted toward lower energies and broader emission bands.

In the subsequent phase of this study, our objective was to establish a correlation between variations in fluorescence spectroscopy among colloidal QD solutions and the optimal conditions of synthesis along with the type of acid employed in the synthesis procedure. The fluorescence decay curves of Ag_2_S QDs (samples **1**–**6**) are illustrated in Figure 1. All six curves were suitably fitted to the biexponential model (eq 1).
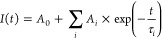
1where *I*(*t*) represents the intensity after time *t*, *A*_0_ is the background intensity, and τ_i_ stand for lifetimes corresponding to *A*_i_ component amplitudes. Decays for all samples could be reasonably well fitted by assuming the presence of two lifetime components (τ_1_ and τ_2_). The fit parameters are listed in Table 2. According to previous studies, the short lifetime can be attributed to core-state recombination, while the longer one can be associated with the surface-related radiative recombination of carriers.^19,20^ In all six tested samples, distinct shorter and longer fluorescence lifetimes are observed, with the former being approximately three to four times shorter than the latter. Noteworthy recurring traits can be discerned among individual pairs of nanoparticles synthesized under identical conditions with the type of acid (2MPA or 3MPA) utilized as the variable parameter. While the actual lifetimes closely resemble each other within individual pairs—for instance, in sample **1**, τ_1_ = 6.45 ns and τ_2_ = 23.33 ns, while in sample **2**, τ_1_ = 6.35 ns and τ_2_ = 22.26 ns—the corresponding lifetime components, in contrast, exhibit significant variation. For sample **1** with 2MPA, the lifetime component *A*_1_ measures 42.65, while for sample **2** with 3MPA, it is 12.48. Within each pair, the reduction in the lifetime component for samples with 3MPA varies from 3.5 times to 2.5 times. The factor that sequentially affects the fluorescent lifetime is most notably observed between **3** and **4**, where the lifetime doubles when transitioning from 2MPA to 3MPA for synthesis at 30 °C for 1 h. Following this trend, a substantial augmentation is evident in the contribution of the longer lifetime component, *A*_2_, in all samples containing 3MPA. The increased prominence of the surface-related longer lifetime component (*A*_2_) could potentially be linked to increase in quantum efficiency^19,20^ as well as a greater contribution of surface-related carrier recombination in the samples containing 3MPA (**2**, **4**, **6**). In the measured fluorescence spectra (Figure 2), the intensities are nearly identical for QDs synthesized for 3 h at 90 °C with Ag:S = 6:1, and for syntheses carried out at lower temperature but the same Ag:S ratio, Ag_2_S-3MPA exhibits higher fluorescence intensity compared to Ag_2_S-2MPA. The same trend is observed for QDs synthesized at 90 °C with Ag:S = 4:1. A consistent observation when comparing spectra of “twin” QDs is a fluorescence peak shift for Ag_2_S-2MPA compared to Ag_2_S-3MPA, ranging from 20 to 40 nm toward longer wavelengths, for 2MPA covered QDs. Additionally, the principle is upheld that in the case of “twin” QDs, the full width at half-maximum (fwhm) of the fluorescence spectrum is wider for those with a protective 3MPA coating. The difference between these fwhm values is dependent on the reaction temperature. At 90 °C, it is a few dozen nanometers, while at 30 °C, it exceeds 70 nm (see Figure S8). Interestingly, our results are contrary to those obtained by the Acar group in their study on CdS quantum dots using 2MPA and 3MPA.^21^ In their case, nanoparticles stabilized with 2MPA exhibited improved emission, longer lifetimes, and a higher contribution from the surface-related longer lifetime component. Their research group attributed the observed distinction to a more effective removal of quenching defects, potentially originating from uncoordinated S^2–^ sites, from the surface of CdS with 2MPA than with 3MPA. However, *ab initio* calculations did not demonstrate significant differences in the electronic structure of 2MPA and 3MPA, both in the gas phase and at the surface, nor did they reveal preferences between these two thioacids for complexing certain or adjacent Cd atoms. The TEM images provided by the Acar research group did not indicate the presence of any self-organization process. In our fluorescence studies, we observed a different feature: the quantum yield (QY) of Ag_2_S-3MPA surpassed that of Ag_2_S-2MPA. However, due to its low values and limitations in detection (measurements up to 900 nm only), the results contain a significant margin of error; therefore, precise comparisons of QYs are not possible; however, we provide approximate data (see Table S1). Moreover, spectroscopic analyses (FT-IR) confirm the absence of significant structural distinctions and no variation in the binding of the branched structures of 2MPA and 3MPA to Ag_2_S. At this stage we note that obtaining improved QY of Ag_2_S QDs may require more in-depth studies of the influence of surface states on the luminescence; however, the usual target is simultaneous improvement of the luminescent properties and nonlinear absorption efficiency. Therefore, we present in the subsequent stage of the study the investigation of nonlinear optical properties of Ag_2_S QDs, including addressing the issue whether the type of acid used has an impact on those properties.

**Table 2:** Fluorescence Short and Log Lifetime (τ) Components, Their Corresponding Amplitudes (*A*), and the Correlation Coefficient (χ^2^) of Ag_2_S QDs in Water

Sample no.	τ_1_ (ns)	*A*_1_ (%)	τ_2_ (ns)	*A*_2_ (%)	τ_av_ (ns)	χ^2^
**1**	6.45	42.65	23.33	57.35	16.13	1.0900
**2**	6.35	12.48	22.26	87.52	20.27	1.0854
**3**	2.47	54.66	13.64	45.34	7.53	1.4650
**4**	3.11	15.97	17.66	84.03	15.36	1.1971
**5**	5.58	44.93	22.32	55.07	14.80	1.2684
**6**	5.94	18.28	21.36	81.72	18.54	1.1386

## IR Spectroscopy

In the case of QDs coated with 2MPA and 3MPA molecules, distinctive shifts of the marker band maxima were detected in the FT-IR spectra (Figures S9 and S10). The investigated samples were prepared under varying conditions (Ag/S ratio, temperature, and time; refer to Table 1). The absorption bands at 2971(m) cm^–1^, 2974(w) cm^–1^ correspond to the antisymmetric stretching vibrations ν_as_ C–H of the 2MPA and 3MPA modifiers, respectively. The symmetric stretching vibrations ν_s_ C–H of the 2MPA and 3MPA generate the bands at 2929(w) cm^–1^, 2941/2937(m) cm^–1^, respectively.^22−24^ One notes an inversion of the intensity of the ν_as_ C–H and ν_s_ C–H bands of the stretching vibrations in 2MPA and 3MPA acids. The FT-IR spectra of Ag_2_S-2MPA/3MPA QDs validate the bonding of MPA through the thiol group as the absence of a ν S–H stretching vibration around 2560 cm^–1^ signifies deprotonation of the SH group upon 2MPA or 3MPA adsorption on Ag_2_S QDs. This confirms that thioacid molecules are bound to the surface of the Ag_2_S QDs via the Ag–S bond.^22−25^

In our discussion regarding the structure of Ag_2_S-2MPA/3MPA QDs, two distinct and robust bands (under identical conditions of temperature and time) hold significance: (i) at 1572, 1578 cm^–1^ (2MPA) and at 1562, 1563 cm^–1^ (3MPA), assignable to the antisymmetric stretching vibration of ν_*as*_ COO^*–*^ group; (ii) at 1399, 1402 cm^–1^ (2MPA) and at 1412, 1408 cm^–1^ (3MPA), originating from the symmetric stretching vibration of ν_*s*_ COO^–^ group.

These frequencies indicate the adsorption of the MPA molecules on the Ag_2_S interfaces in the ionic form with a free terminal group COO^–^.^22,24,25^ No significant disparity is observed between the antisymmetric and symmetric stretching vibrations of carboxylates, indicating diverse binding modes of 2MPA or 3MPA.

A noteworthy distinction lies in the intensity ratio for antisymmetric and symmetric stretching vibrations COO^–^ (ν_*as*_ COO^–^ /ν_*s*_ COO^–^), with the ratio being higher for Ag_2_S-3MPA QDs. This feature results from the altered symmetry of the vibration mode of the two acids, particularly 3MPA, upon their adsorption on a solid substrate, affecting the organization of Ag_2_S QD structures.

Furthermore, the weak bands around 928 cm^–1^ correspond to ν S–C stretching vibrations, while the medium bands at 690 cm^–1^ (2MPA) and 670 cm^–1^ (3MPA) originate from ν Ag–S stretching vibrations as well.^23^

## NLO Measurements

There are two well-known experimental techniques for quantitative determination of the nonlinear absorption properties of a material. The popular two-photon excited fluorescence technique^26,27^ relies on measuring the fluorescence signal following two-photon (or multiphoton) excitation, typically using an appropriate reference sample. The Z-scan technique,^28,29^ on the other hand, allows the determination of two-photon (and multiphoton) absorption cross-sectional values for nonfluorescent molecules, and it can be used for the determination of factors describing both the nonlinear absorption of various types (including two-photon absorption, absorption saturation, and reverse saturation) and the nonlinear refraction.

**Figure 3:**
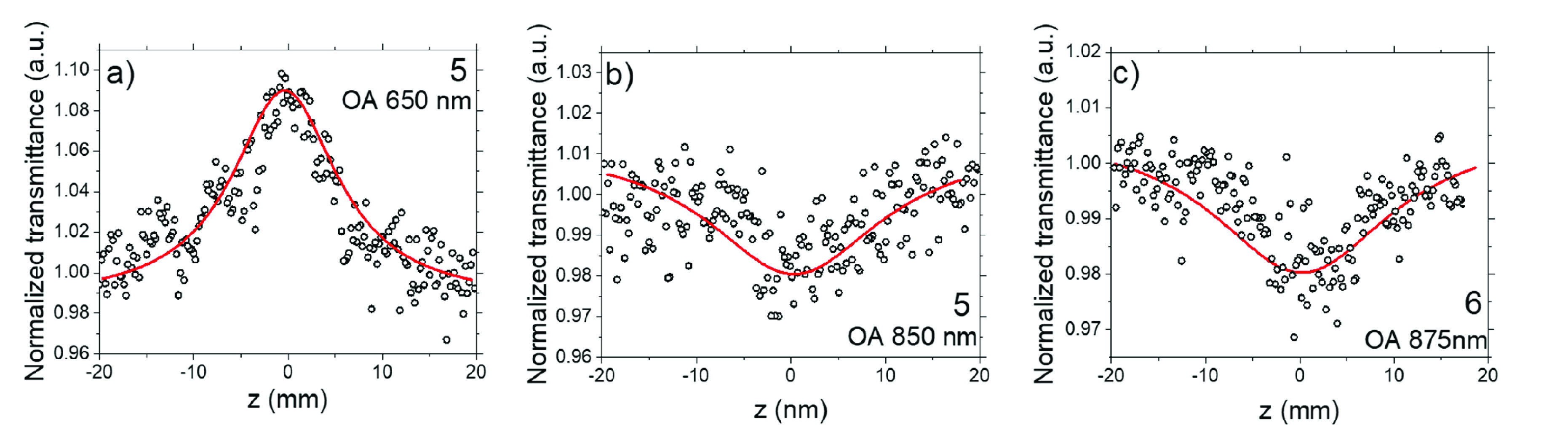
Representative open aperture Z-scan traces of (a) sample **5** at 650 nm indicating the saturation of 1PA, (b) sample **5** at 850 nm (fitting was performed using an equation describing the 2PA phenomenon), and (c) sample **6** at 875 nm, a dip in the OA trace at the focus is seen, indicating 2PA.

Among the synthesized samples, we chose those exhibiting the highest stability and the most interesting optical properties for further investigation of their nonlinear optical characteristics. In our study, we conducted Z-scan experiments on two representative colloidal quantum QDs with 2MPA sample **5** and 3MPA sample **6**, whose detailed characteristics are presented above. While many literature Z-scan experiments are limited to single wavelengths, we have performed a spectrally resolved study using excitation in a broad range of the Vis and NIR wavelengths, covering the regions of weak to moderate one-photon absorption. Representative OA Z-scan traces for samples **5** and **6** are depicted in Figure 3. At shorter wavelengths, only an increase in the transmittance at the focus of the beam was observed (Figure 3a), suggesting that saturable absorption dominates over any multiphoton processes. However, at longer wavelengths, a dip in the optical absorption traces at the focus is observed, indicating the presence of a 2-PA process (Figure 3b,c).

In order to perform the quantitative analysis of the results we used equations derived by Sheik-Bahae et al.^30^ introducing additional modifications caused by the saturation of 1PA. Generally speaking, in the samples we have investigated, where both 2PA and 1PA saturation occur (Figure 3), it can be assumed that the process of light absorption by the nanoparticles can be represented using the equation
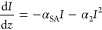
2where the usual one-photon absorption coefficient is substituted with the saturable 1PA coefficient α_SA_. Several saturable absorption models have been described in the literature;^31,32^ however, in this work, it has been sufficient to apply the equation for the saturable 1PA which takes place within a homogeneously broadened absorption band (Figure 3a):
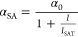
3

**Figure 4:**
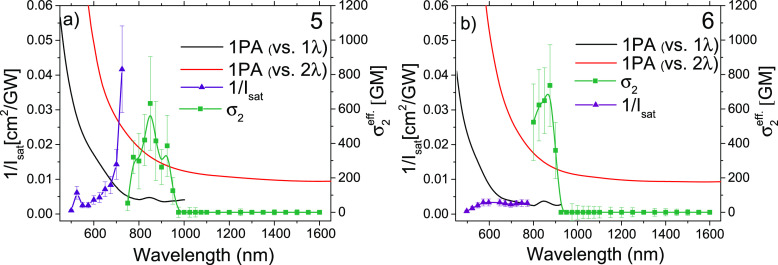
Nonlinear absorption strength at different wavelengths in the infrared regions and the reciprocate of saturation intensity in the 1PA in the visible region for **5** (a) and **6** (b) in water. The σ_2_^eff^ values are plotted as green squares for a corresponding wavelength (the green solid line is used to guide the eye). The reciprocals of saturation intensity of 1PA are plotted as violet filled triangles (the violet line is used to guide its distribution). The black line represents the 1PA spectra plotted vs 1λ, and the red line represents the same 1PA spectra plotted vs 2λ.

For shorter wavelengths, ranging from 500 to 725 nm for sample **5**, and up to 775 nm for sample **6**, only 1PA saturation is observed. It is important to note that the reciprocals of one photon saturation intensity are significantly higher for sample **5** than for sample **6**. The investigated samples had nearly identical concentrations and were synthesized under the same conditions; thus, the only parameter that could account for this difference is the acid used for synthesis. From our previous studies on gold nanoparticles,^28,33^ we know that the saturation intensity value is a function of the photon energy, the cross-section for one-photon absorption, and the lifetime of the excited state. Referring to the measurements of fluorescence lifetime conducted by us, it can be noted that sample **5** prepared using 2MPA acid exhibits a larger contribution of a core-state recombination, while sample **6** is dominated by the surface-related radiative recombination of carriers. One can come to the conclusion that the observed differences in the reciprocals of saturation intensity of 1PA may have their roots in these variations; however, in order to prove such a thesis, it would be necessary to make additional time-resolved measurements, at different powers of excitation light. At longer wavelengths in the infrared region samples **5** and **6** show characteristic minima at the focal points for Z-scans (Figure 3 b,c) indicating 2PA; however, since some contribution from excited state absorption cannot be excluded there, we refer to the determined cross section values as σ_2_^eff^ (Figure 4).

In the regions 750–950 nm (for **5**) and 800–900 nm (for **6**) the studied compounds possess the highest σ_2_ values equal to 632 ± 270 GM (at 850 nm) for **5** and 772 ± 100 GM (at 875 nm) for **6** determined at the intensity of 85–133 GW/cm^2^. At this stage, it is worthwhile to compare the nonlinear absorption strengths of the investigated QDs to those of other types of nanomaterials. This can be done by employing various merit factors.^34^ When dealing with 2PA, it is useful to normalize the σ_2_ values of different species by dividing them by their molar masses (M). The value of σ_2_/M for sample **5** at 850 nm is equal to 0.013 (GM mol g^–1^), while that for sample **6** at 875 nm is equal to 0.012 (GM mol g^–1^). This leads us to a conclusion that the type of acid used for Ag_2_S QD synthesis does not affect the efficiency of 2PA. The comparison of the values of the nonlinear optical parameters obtained by us with the data available in the literature is not straightforward. The closest to our research is the work of Guo at al.^35^ Their studies were conducted on 4 nm quantum dots using 60 fs pulses of 800 nm radiation, and the obtained nonlinear absorption coefficient, α_2_, value was 8.8 × 10^–8^ cm W^–1^; unfortunately, there is no available data on the value of σ_2_. On the other hand, one can compare our σ_2_/M values with those for well-studied CdS QDs, for which this value is 0.038.^36^ Taking into account the significantly lower toxicity of our material, as well as the presence of one-photon saturable absorption and 2PA at various wavelengths, we believe that silver-sulfide-based CDs hold great potential for various applications in laser technologies and photomedicine.

Finally, we present the spectral dependences of the real (refractive) part of the complex hyperpolarizability Re(γ) (Figure S11) calculated from the Z-scan measurements on samples **5** (with 2MPA) and **6** (with 3MPA). From our measurements and calculations, it follows that sample **6** (with 3MPA) has larger values of Re(γ) and smaller reciprocals of one photon saturation intensity, compared to sample **5** prepared with 2MPA. The representative closed-aperture (CA) z-scan traces are presented in Figure S12. We conclude that while the type of acid used as the ligand does not seem to affect the efficiency of 2PA, this may not be valid in the case of one-photon absorption saturation and nonlinear refraction properties, which may both depend on the one-photon absorption properties, apparently differing for the two investigated samples in the range 600–800 nm.

In summary, this study aimed to explore Ag_2_S QDs as less toxic alternatives to traditional near-infrared-emitting QDs, such as those based on cadmium, mercury, and lead, for effective 2PA and emission within the NIR range. Indeed, we found that the Ag_2_S QDs do possess reasonably high σ_2_ values (632 ± 270 GM for Ag_2_S-2PA QDs and 772 ± 100 GM for Ag_2_S-3PA QDs) at wavelengths in the vicinity of 800 nm. Considering that the QDs were synthesized in water following a procedure that ensures their long-term stability, this further enhances their applicability, especially in biological contexts. Therefore, one should address the various opportunities to improve both the NLO properties of Ag_2_S based materials and their luminescent properties, through the avenues such as modification of nanoparticle shapes (see ref (37)), changing of their surface ligands, and doping with other metals, which we will pursue in future research.^38−41^ We note that although the research was conducted over a wide range of wavelengths (500–1600 nm), no range was detected in which the studied samples exhibited three-photon absorption. On the other hand, the wavelength range of useful 2PA seems to be limited by the presence of a relatively long tail of one-photon absorption in the NIR. Modification of synthesis conditions may lead to improvements in such absorption features. Besides their 2PA properties, the nonlinear refraction and one-photon absorption saturation exhibited by Ag_2_S QDs may also need further attention, in view of possible applications in data storage and other optical devices.

## Experimental Methods

### Synthesis

For the Ag_2_S QDs synthesis, all chemicals were procured from Merck except for 2-mercaptopropionic acid (2MPA), which was acquired from Thermo Fisher Scientific. The synthesis procedure followed the method outlined by the Acar group.^2^ In two separate instances, solutions were prepared using 75 and 25 mL of deionized water for silver nitrate (V) with mercaptopropionic acid (2MPA or 3MPA, referred to as MPA) and sodium sulfide, respectively. The reagents were deoxygenated, evacuated under vacuum, and stored under a nitrogen atmosphere. The setup was also prepared in this nitrogen environment. A three-neck flask was utilized, and 75 mL of water was added, followed by the introduction of MPA to stabilize the pH at 7.5. Subsequently, silver nitrate (V) was introduced, and the pH was once again adjusted to 7.5 by using acetic acid and sodium hydroxide. Heating was initiated in an oil bath with a reflux condenser, maintaining a nitrogen atmosphere at either 30 or 90 °C. Once the desired temperature was reached, a solution of sodium sulfide dissolved in 25 mL of water was gradually added dropwise, ensuring a Ag:S ratio of either 6:1 or 4:1. Following the complete injection of the Na_2_S solution, the synthesis, lasting 1 or 3 h, commenced with continuous stirring of the reaction mixture. Upon synthesis completion and cooling of the product, the resulting solution was filtered through a 0.22 μm syringe filter. Subsequently, the solution was centrifuged using Vivaspin 3 kDa centrifugal filtration units, transferred into a dark container, and stored in a refrigerator for future use.

### Characterization Methods

The Malvern Zetasizer Ultra Red instrument was employed for measuring the hydrodynamic size of nanoparticles within colloidal solutions as well as determining the zeta potential through the application of electrophoretic light scattering methodology. This was accomplished by using the Smoluchowsky model.

Powder X-ray diffraction (XRD) patterns were recorded using a PANalytical AERIS diffractometer. The measurement was performed in the 2θ range of 20–70°, using Cu Kα_1_ radiation (λ = 1.5406 Å) with the counting time of 300 s and the step of 0.02. The reference pattern was taken from Joint Committee on Powder Diffraction Standards (JCPDS) card no. 14-0072 (Ag_2_S).

The sizes and arrangement of the quantum dots (QDs) were characterized by using an FEI Tecnai G^2^ 20 X-TWIN Transmission Electron Microscope. The solutions were deposited onto TEM grids, subjected to drying, and subsequently measured. The absorption spectra of the solutions were determined using a Cary 5000 spectrophotometer.

The vibrational spectral measurements were performed in the Laboratory of Vibrational Spectroscopy belonging to the Faculty of Chemistry at the Wrocław University of Science and Technology. ATR FT-IR spectra of 2MPA/3MPA functionalized Ag_2_S QDs were collected using a Bruker Vertex 70v Fourier transform infrared spectrometer equipped with an air-cooled DTGS detector and diamond attenuated total reflection infrared cell at 2 cm^–1^ resolution and using 64 scans in the middle-infrared (4000–400 cm^–1^) region at room temperature. The procedure was conducted by applying a drop of samples to the diamond plate of the ATR accessory.

Emission spectra and luminescence decay traces were captured by using an FLS1000 Fluorescence Spectrometer from Edinburgh Instruments Ltd. In the case of fluorescence measurements, a 450 W continuous xenon arc lamp served as the excitation source, while luminescence decay traces were obtained using a picosecond pulsed diode laser EPL-450 nm from Edinburgh Instruments. The setup utilized TMS302-X double-grating excitation and emission monochromators with a focal length of 325 mm. The luminescence signal was detected using a Hamamatsu R928P high-gain photomultiplier detector, which was thermoelectrically cooled to −22 °C.

The intensity decay curves were fitted using exponential equations and Fluoracle software. All luminescence experiments were conducted with the samples placed in 1 cm quartz cuvettes.

Simultaneous closed- and open-aperture Z-scan measurements were conducted using a setup and methodologies previously detailed in the literature.^33,37,40−42^ Briefly, approximately 55 fs laser pulses at a 1 kHz repetition rate were generated by an Astrella Ultrafast regenerative amplifier, functioning as the 800 nm pump for a TOPAS-PRIME parametric amplifier, which facilitated wavelength tuning across the range of 500–1600 nm. Subsequently, the laser beam was focused to a focal spot with a beam waist of *w*_0_ = 26–73 μm using a lens. The calculated intensity at the focal point of the arrangement ranged from 100 to 390 GW/cm^2^. The employed pulse energies were around 1 μJ, which corresponded to the average power of approximately 1 mW. For signal detection, three silicon (for Vis region) or InGaAs (for NIR region) photodiodes were deployed: for reference, open-aperture, and closed-aperture power measurements. These signals were then digitized with a National Instruments PCI-6143 I/O card with simultaneous sampling across all channels synchronized with the laser. The acquired data were subsequently transferred to a computer utilizing custom LabVIEW software. As consistent with previous work conducted in our laboratory, the Z-scan data acquired from colloidal nanoparticle solutions at different wavelengths^9,28,33,36,43,44^ were calibrated against closed-aperture measurements performed on a 3 mm thick silica plate. The concentrations of the nanoparticle samples in water were 11.1 mg/mL for sample **5** and 11.2 mg/mL for sample **6**.

## Supporting Information Available

XRD patterns of the prepared Ag_2_S QDs after drying; UV–vis absorption spectra of Ag_2_S QDs colloidal solutions **1**–**6**; calculated values of quantum yield for the investigated samples; TEM images of Ag_2_S QDs **1**–**6**; dynamic light scattering (DLS) measurements of samples **3** and **4**; normalized fluorescence emission spectra of samples **1**–**6**; FT-IR spectra of samples **3**–**6**; real parts of the second hyperpolarizability (γ) of sample **5** and **6**; representative closed-aperture (CA) z-scan traces of solvent and samples **5** and **6** at 550 nm (PDF)
