# Supplementary material for: Third-Order Nonlinear Optical Properties of Aqueous Silver Sulfide Quantum Dots
Source: J Phys Chem Lett. 2023 Dec 6;14(49):11117–24. doi: 10.1021/acs.jpclett.3c02820 (PMC10755751; doi:10.1021/acs.jpclett.3c02820)
Supplement: Supplementary file 1 — jz3c02820_si_001.pdf [file jz3c02820_si_001.pdf]

Supporting Information

Third-Order Nonlinear Optical Properties of Aqueous Silver Sulfide Quantum Dots

Marta Gordel-Wójcik,[a]* Magdalena Malik,[b] Agnieszka Siomra,[c] Marek Samoć,[c], Marcin Nyk[c]

[a]Faculty of Chemistry, University of Wrocław, 14.p F. Joliot-Curie Street, 50-383, Wrocław, Poland

[b]Faculty of Chemistry, Wrocław University of Science and Technology, Wyb. Wyspianskiego 27, 50-370, Wrocław, Poland

[c]Institute of Advanced Materials, Faculty of Chemistry, Wrocław University of Science and Technology, Wyb. Wyspiańskiego 27, 50-370, Wrocław, Poland

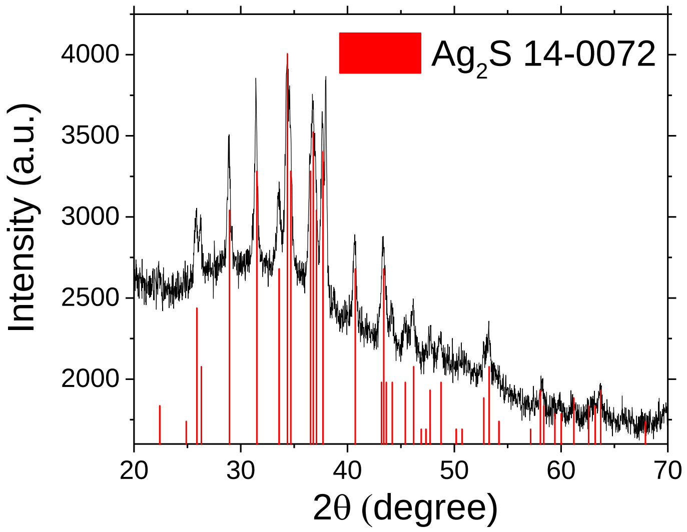

Fig. S1 *XRD patterns of the prepared Ag_2S QDs after drying (black line), JCPDS Card No. 14-0072 (Ag_2S) (red line).*

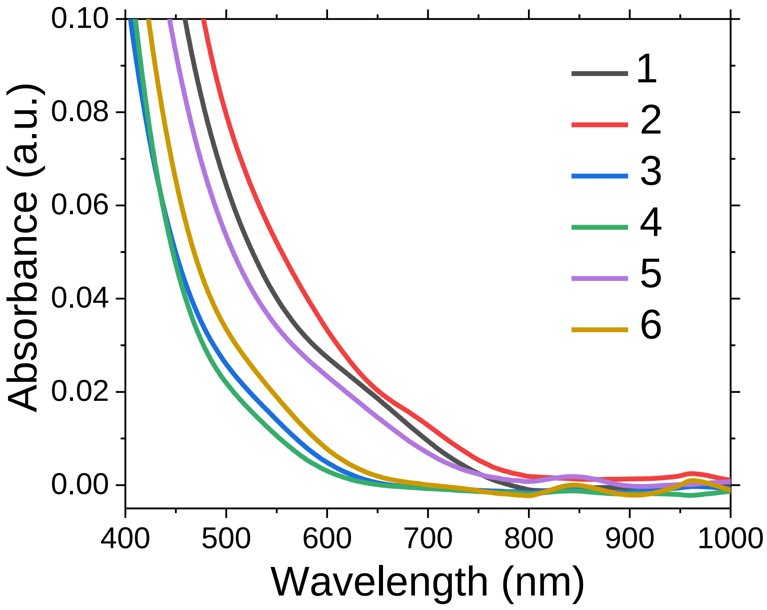

Fig.S2 UV-Vis absorption spectra of Ag_2S QDs colloidal solutions ***1***-***6***.

Table S1 Calculated values of quantum yield (QY) for the investigated samples, in reference to indocyanine green ICG in DMSO.

Sample name	1	2	3	4	5	6
QY %	0.10	0.55	0.10	0.50	0.12	0.66

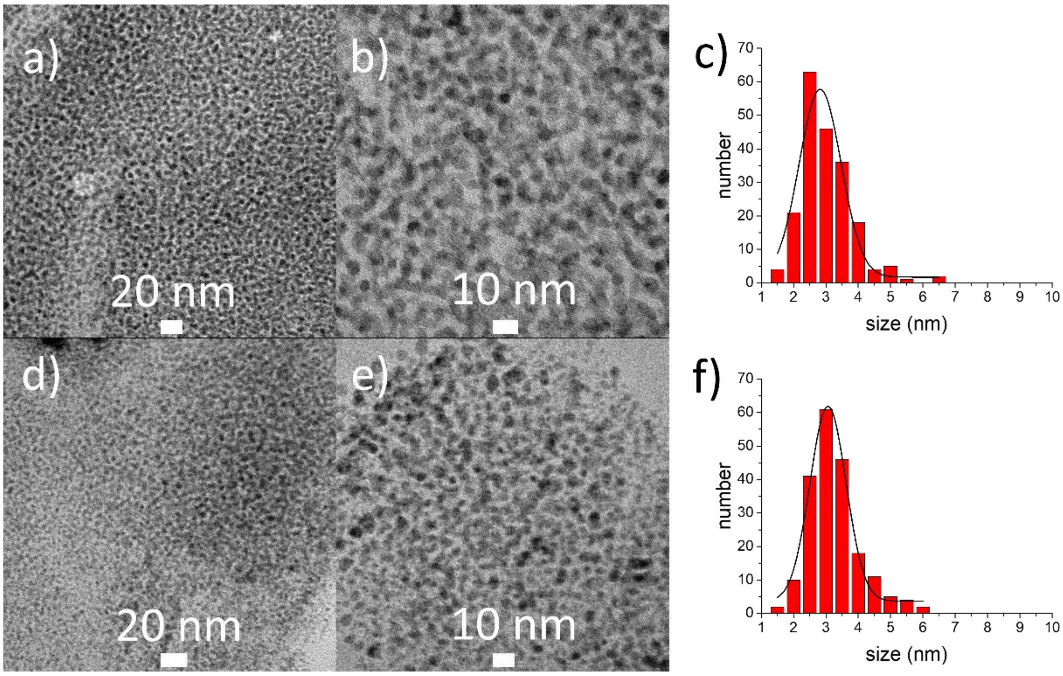

*Fig. S3 TEM images of Ag_2S QDs **1** (a,b) and **2** (d,e), along with the statistical size distribution of sample **1** (c) and sample **2** (f). The examined samples did not exhibit self-assembly following their deposition onto the TEM grid and subsequent drying. These samples were prepared with the Ag to S ratio of four at a temperature of 90°C. In both cases, the initial solution had a pH of 8.9 for 2MPA QDs and 8.8 for 3MPA QDs, respectively.*

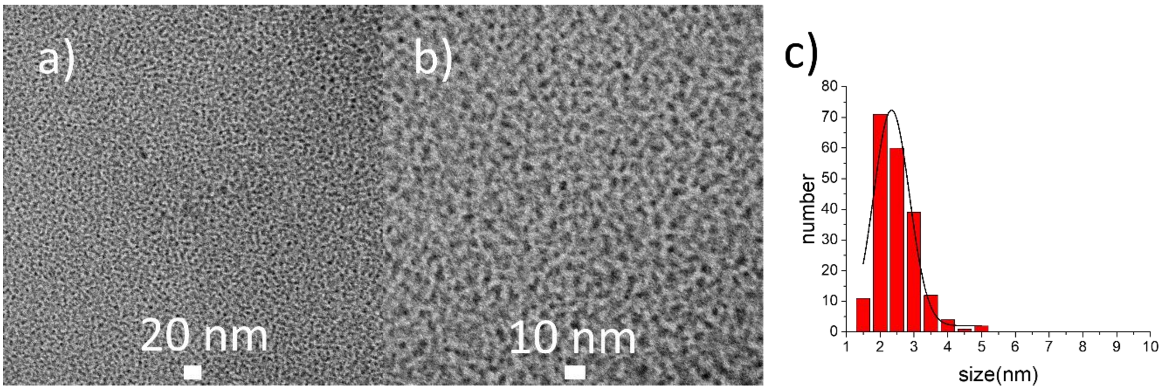

*Fig. S4 TEM images of Ag_2S QDs for sample **3** (a,b) and the corresponding statistical size distribution*

(c). The sample synthesized at 30°C using 2MPA exhibited no signs of self-assembly.

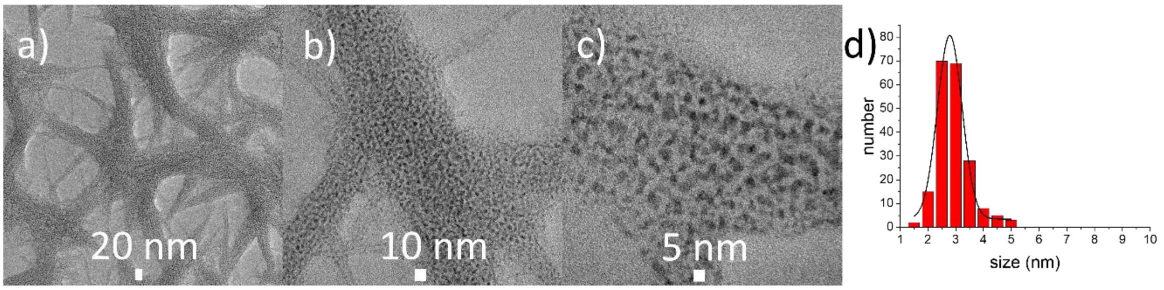

*Fig. S5 TEM images of Ag_2S QDs for sample **4** (a,b,c) at different magnifications, along with the corresponding statistical size distribution (d). Moving from left to right, a gradual increase in magnification reveals the self-assembly of QDs prepared at 30°C using 3MPA, forming elongated structures with varying diameters.*

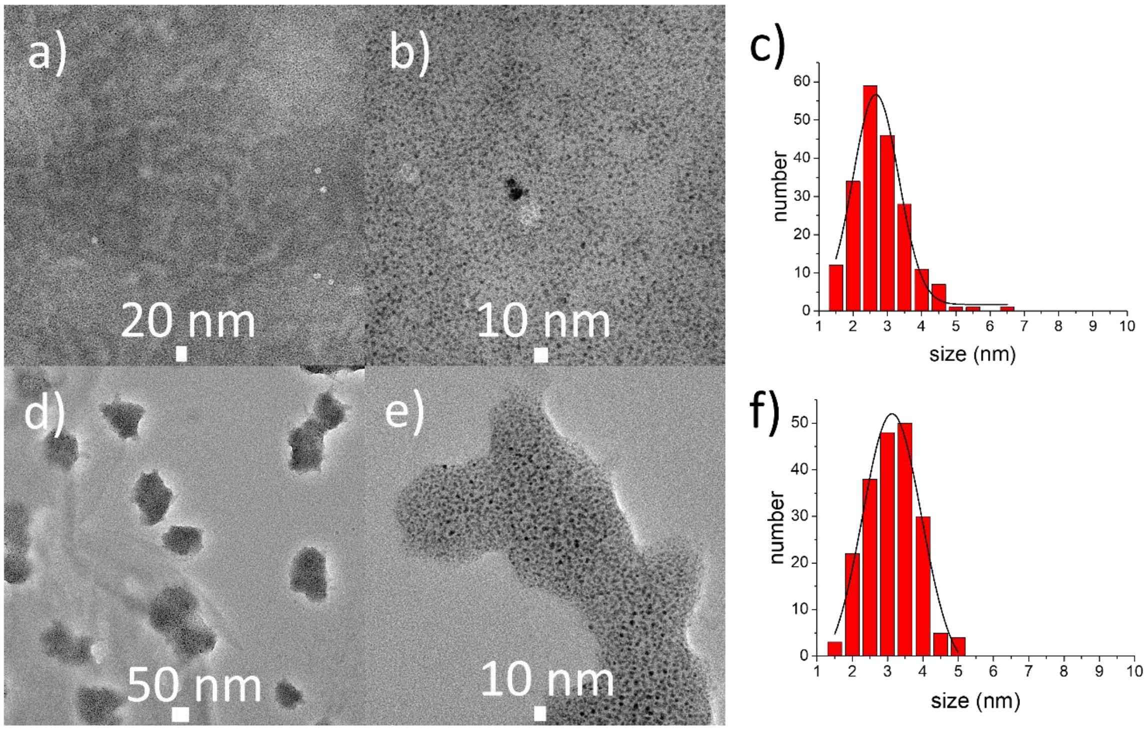

*Fig. S6 TEM images of Ag_2S QDs for sample **5** (a,b) and sample **6** (d,e), along with the statistical size distribution for sample **5** (c) and sample **6** (f). These TEM images illustrate the synthesis of QDs with the silver-to-sulfide ratio of **6**. While the sample prepared using 2MPA does not exhibit self-assembly (sample **5**), the sample prepared under the same conditions but using 3MPA demonstrates the process of self-assembly upon deposition onto a TEM grid and subsequent drying. Sample **6** forms irregular-shaped structures, differing from the elongated structures observed in sample **4**, self-assembling into more spherical entities.*

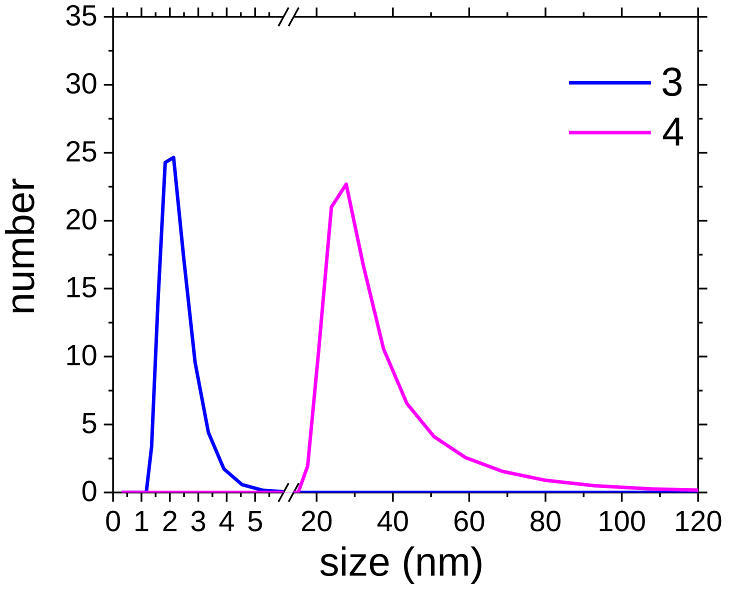

*Fig. S7 Dynamic light scattering (DLS) measurements conducted to determine the hydrodynamic diameter of colloidal QDs in samples **3** and **4**. The QDs hydrodynamic diameter (sample **3**, blue line) measured using this method is in agreement with the dimensions observed on TEM images (Fig. S4). Conversely, the measurement for sample **4** demonstrates a wide band that corresponds to nanoparticles with larger hydrodynamic diameters, potentially indicating the formation of three-dimensional spatial structures, as seen on the TEM image (Fig. S5).*

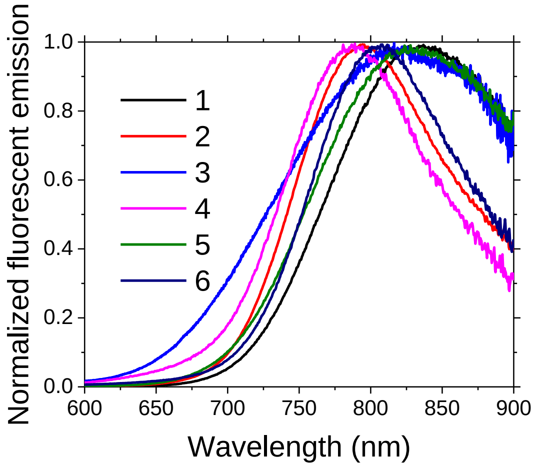

*Fig. S8 Normalized fluorescence emission spectra of the samples measured under excitation at 450 nm. The variation in maximum absorption for each pair of 'twin' nanoparticles is approximately 40 nm for pairs **1** and **2**, around 30 nm for pairs **3** and **4**, and about 20 nm for pairs **5** and **6**.*

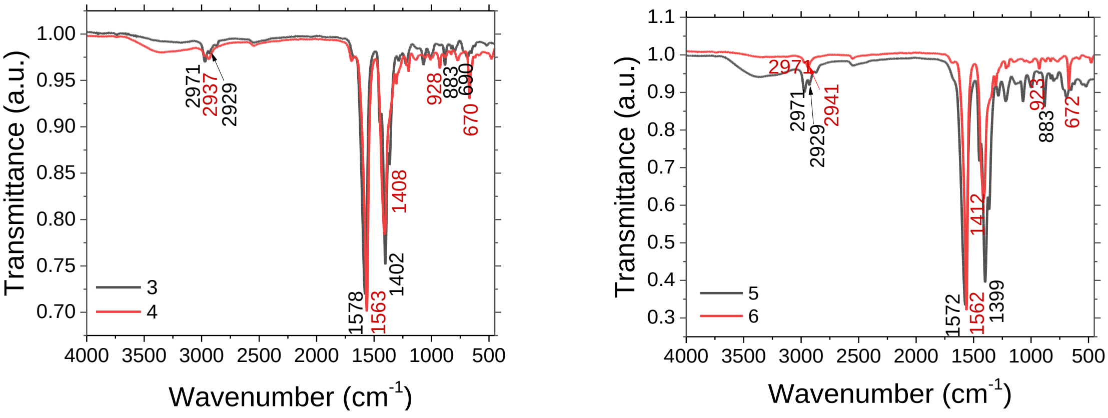

*Fig. S9. The comparison of the FT-IR spectra of Ag_2S-2MPA QDs (**3**, **4**) – left side and Ag_2S-3MPA QDs (**5**, **6**) – right side; synthesis parameters are given in Tab. 1.*

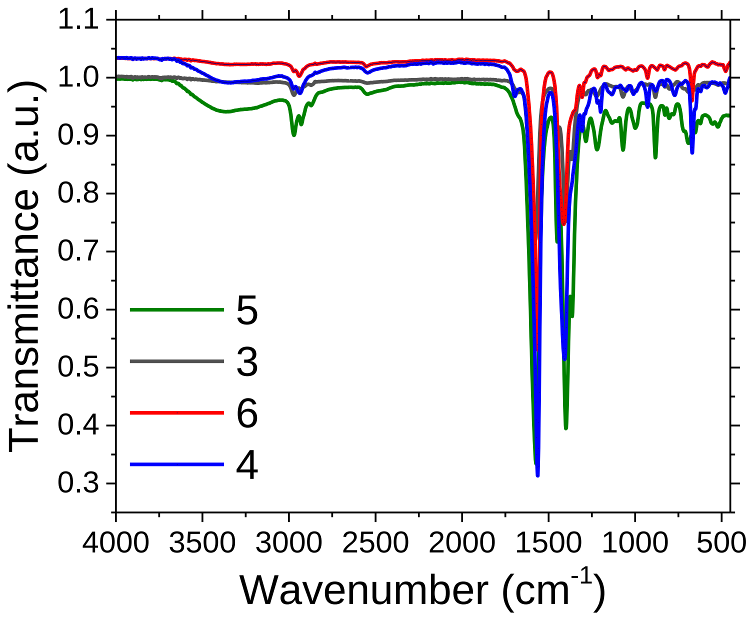

Fig. S10. Comparison of the FT-IR spectra of Ag_2S/2MPA and Ag_2S/3MPA QDs whose synthesis was carried out under different conditions.

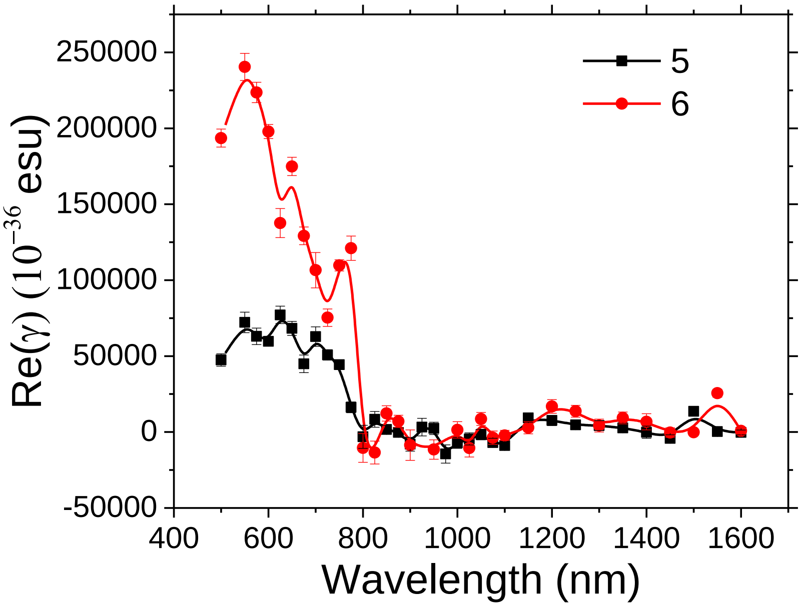

*Fig. S11 Real parts of the second hyperpolarizability (γ) of sample **5** (with 2-MPA) and **6** (with 3-MPA). Values correspond to individual nanoparticles. Lines connecting the experimental points are meant to guide the eyes.*

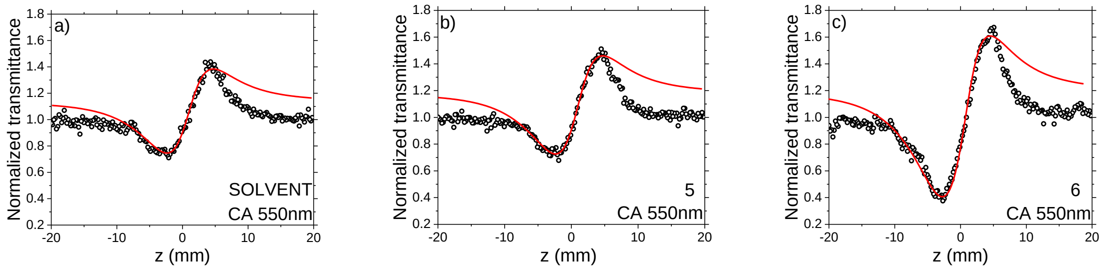

Fig. S12 Representative closed-aperture (CA) z-scan traces of a) solvent b) sample 5 c) sample 6 at 550 nm.
